# Transurethral flexible ureteroscopic incision and drainage with holmium laser in the treatment of parapelvic renal cysts: A retrospective study

**DOI:** 10.1590/S1677-5538.IBJU.2022.0142

**Published:** 2022-05-30

**Authors:** Xiangjun Meng, Qiwu Mi

**Affiliations:** 1 Dongguan People's Hospital Department of Urology Dongguan Guangdong China Department of Urology, Dongguan People's Hospital, Dongguan, Guangdong, P.R. China

**Keywords:** Kidney, Ureteroscopy, Lasers, Solid-State

## Abstract

**Background:**

We aimed to investigate the clinical efficacy and safety of transurethral flexible ureteroscopic incision and drainage with holmium laser in the treatment of parapelvic renal cysts.

**Materials and Methods:**

Between October 2017 and April 2021, the clinical data of 65 patients with parapelvic renal cysts were evaluated retrospectively. Thirty-one patients with parapelvic cysts (Group 1) underwent a transurethral flexible ureteroscopic incision and drainage with a holmium laser, whereas the other 34 patients (Group 2) underwent retroperitoneal laparoscopic unroofing. The patients' clinical features were documented. The surgery time, intraoperative blood loss, hospitalization time, complications and cyst size were recorded and statistically assessed one year following the procedure.

**Results:**

All of the patients were successfully treated with flexible ureteroscopic incision and drainage or retroperitoneal laparoscopic unroofing. In terms of clinical parameters, such as age, gender, BMI, location, cyst size, and Bosniak classification of renal cysts, no statistically significant difference was detected between Groups 1 and 2. Compared to the control group (Group 2), Group 1 demonstrated a shorter surgery duration, less intraoperative blood loss, and a shorter hospital stay (p < 0.001). However, no significant differences in complications and cyst size were observed between the two groups one year after the surgery (p > 0.05).

**Conclusions:**

Transurethral flexible ureteroscopic incision and drainage with holmium laser in the treatment of parapelvic renal cysts has obvious advantages over traditional surgery, and is worthy of advancement and application, but its long-term effect needs further follow-up studies.

## INTRODUCTION

Renal cysts are quite prevalent in the urinary system. Renal cysts affect 50% of adults over the age of 50, and 66 percent of adults will have developed renal cysts by the age of 80, according to studies (1). Parapelvic renal cysts are associated with a special type of renal cystic disease (2), and non-hereditary cysts that develop adjacent to the renal pelvis are especially serious. Renal cysts originate from the renal sinus, around the renal pelvis or renal sinus as parapelvic cysts, according to Kiryluk and Gupta (3).

The clinical symptoms of parapelvic cysts are usually atypical. Parapelvic cysts can be diagnosed by ultrasonography, enhanced computed tomography (CT), magnetic resonance imaging and other imaging examinations. Parapelvic cysts are usually benign lesions with slow progression. If the cyst is tiny, the patient has no symptoms, no complications are observed, and imaging examination reveals no evident renal pelvic compressions, conservative surveillance and regular follow-up are recommended. Parapelvic cysts may compress the renal arteries or renal pelvis due to their unique location, resulting in hypertension, hydronephrosis, and other symptoms. When symptoms appear as a result of compression, aggressive therapy should be initiated.

Laparoscopic renal cyst unroofing is the preferred treatment for parapelvic cysts (4). With the development of minimally invasive technology, Yu et al. (5) reported that incision and drainage under ureteroscopy were performed to treat parapelvic cysts. How ever, just a few studies compared the procedure to laparoscopic renal cyst unroofing. In the treatment of parapelvic cysts, we believe that transurethral flexible ureteroscopic incision and drainage offers greater advantages than laparoscopic renal cyst unroofing. Therefore, this study aims to report the clinical efficacy and safety of transurethral flexible ureteroscopic incision and drainage, compared with retroperitoneal laparoscopic unroofing in the treatment of parapelvic renal cysts.

## MATERIALS AND METHODS

The study was authorized by the Ethics Committee of Dongguan People's Hospital (Dongguan, China) (Ethics approval no.: XJS2018-009). Individual participants agreed to publish informed consent forms with detailed information and provided signed informed consent.

Between October 2017 and April 2021, 65 patients with parapelvic renal cysts treated by transurethral flexible ureteroscopic incision and drainage or retroperitoneal laparoscopic unroofing were retrospectively enrolled in this study. Among them, 31 patients with parapelvic cysts (Group 1) underwent a transurethral flexible ureteroscopic incision and drainage with holmium laser, and the other 34 (Group 2) underwent retroperitoneal laparoscopic unroofing. Surgeries were performed according to normal methods by a surgeon with ten years of experience. The patients’ clinical characteristics are reported in Table-1. Preoperative intravenous urography, ultrasonography and CT were performed to diagnose parapelvic cysts. As needed, retrograde pyelography was performed. Patients with parapelvic cysts >3 cm were included in this study. Patients with parapelvic cysts that were suspected to be malignant according to CT, were excluded. In addition, those with an uncontrolled urinary tract infection, urethral or ureteral stricture, hemorrhagic diseases and cardiopulmonary insufficiency were excluded.

**Table 1:** Comparison of clinical and perioperative factors between flexible ureteroscope incision (Group 1) and retroperitoneal laparoscopic unroofing (Group 2).

Variable		Group 1	Group 2	P value
Patients (n)		31	34	NA
Age (years)		47.6 ±8.7	46.8 ±7.8	0.701
**Gender (n)**				0.73
	Male	15 (48.4)	15 (51.6)	
	Female	16 (44.1)	19 (55.9)	
*BMI (kg/m^2^)		23.8 ±2.2	24.1 ±2.0	0.551
**Location**				0.271
	Right	14 (46.2)	20 (58.8)	
	Left	17 (54.8)	14 (41.2)	
※Cyst size (cm)		5.3 ±0.9	5.1 ±0.9	0.333
**Bosniak classification of renal cysts**				0.336
	Bosniak I	31 (100)	33 (97.1)	
	Bosniak II	0 (0)	1 (2.9)	
Surgery duration (min)		30.1 ±4.3	54.4 ±6.4	<0.001
Blood loss (mL)		5.5 ±1.7	59 ± 9.9	<0.001
Length of hospitalization (days)		4.5 ± 0.8	5.6 ±0.9	<0.001
Cyst size at 1 year, postoperatively (cm)		1.0 ±0.9	0.6 ±0.6	0.106
Complications (n)		1	1	0.947

Data are presented as the mean ± SD or number (percent);

NA indicates not applicable;

* BMI indicates body mass index;

※ Cyst size = the diameter of the stone based on preoperative CT scanning.

Preoperatively, urine analysis, urine culture, and serum biochemical tests were performed in all patients. Patients with infection were not submitted to the procedures until the infection was controlled. All patients were administered a dose of prophylactic antibiotics 30 min preoperatively. In an outpatient department, all patients were followed up at 1, 3, and 12 months postoperatively. Ultrasonography and CT scans were used in the follow-up assessment.

### Surgical protocol

**Figure 1:**
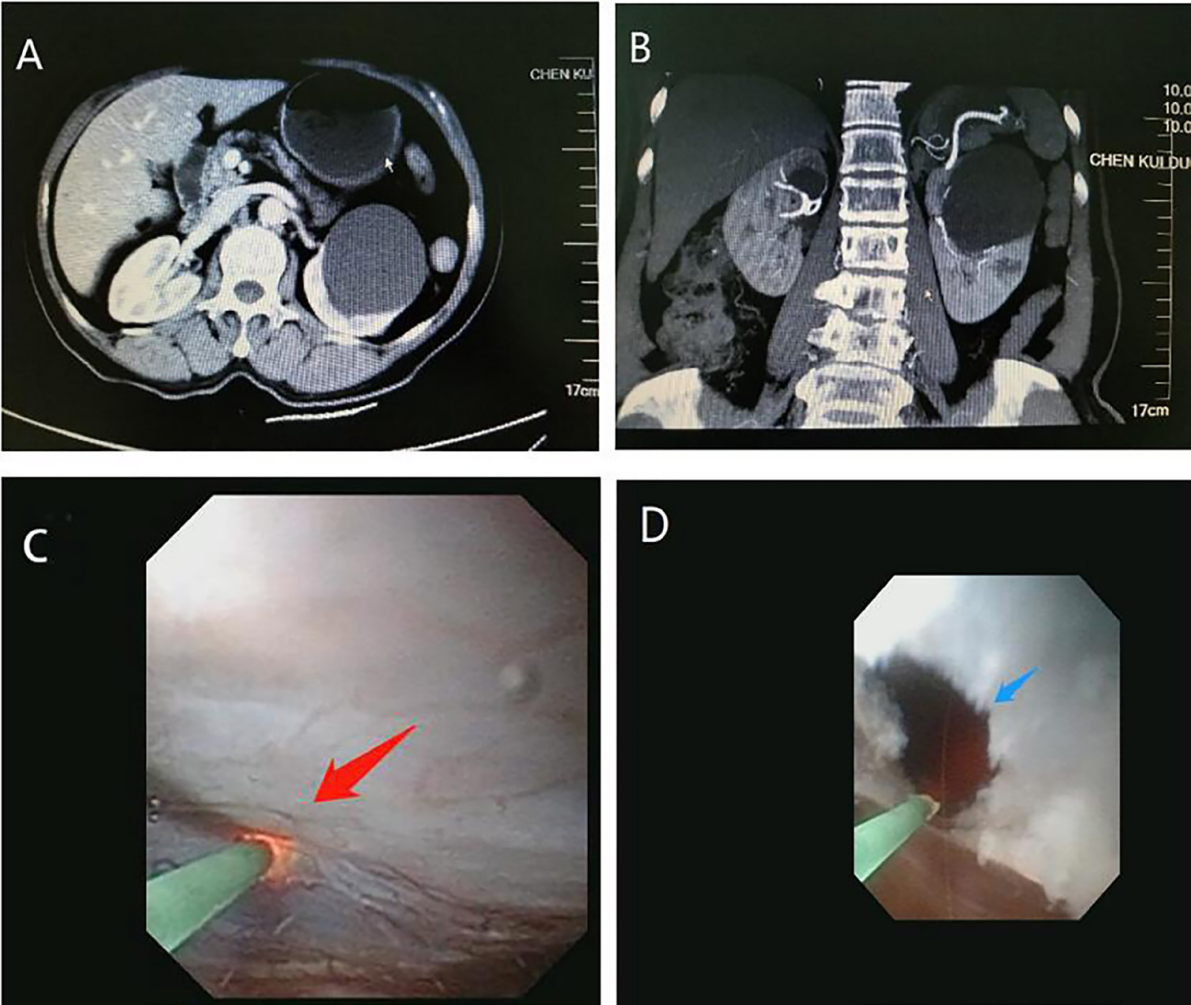
A 67-year-old woman underwent a transurethral flexible ureteroscope incision and drainage. The maximum intensity projection image showed parapelvic cyst in left kidney (A and B). The typical wall (red arrow) of parapelvic cyst looked transparent (C). The image of parapelvic renal cyst after the flexible ureteroscope incision (blue arrow) and drainage (D).

**Figure 2:**
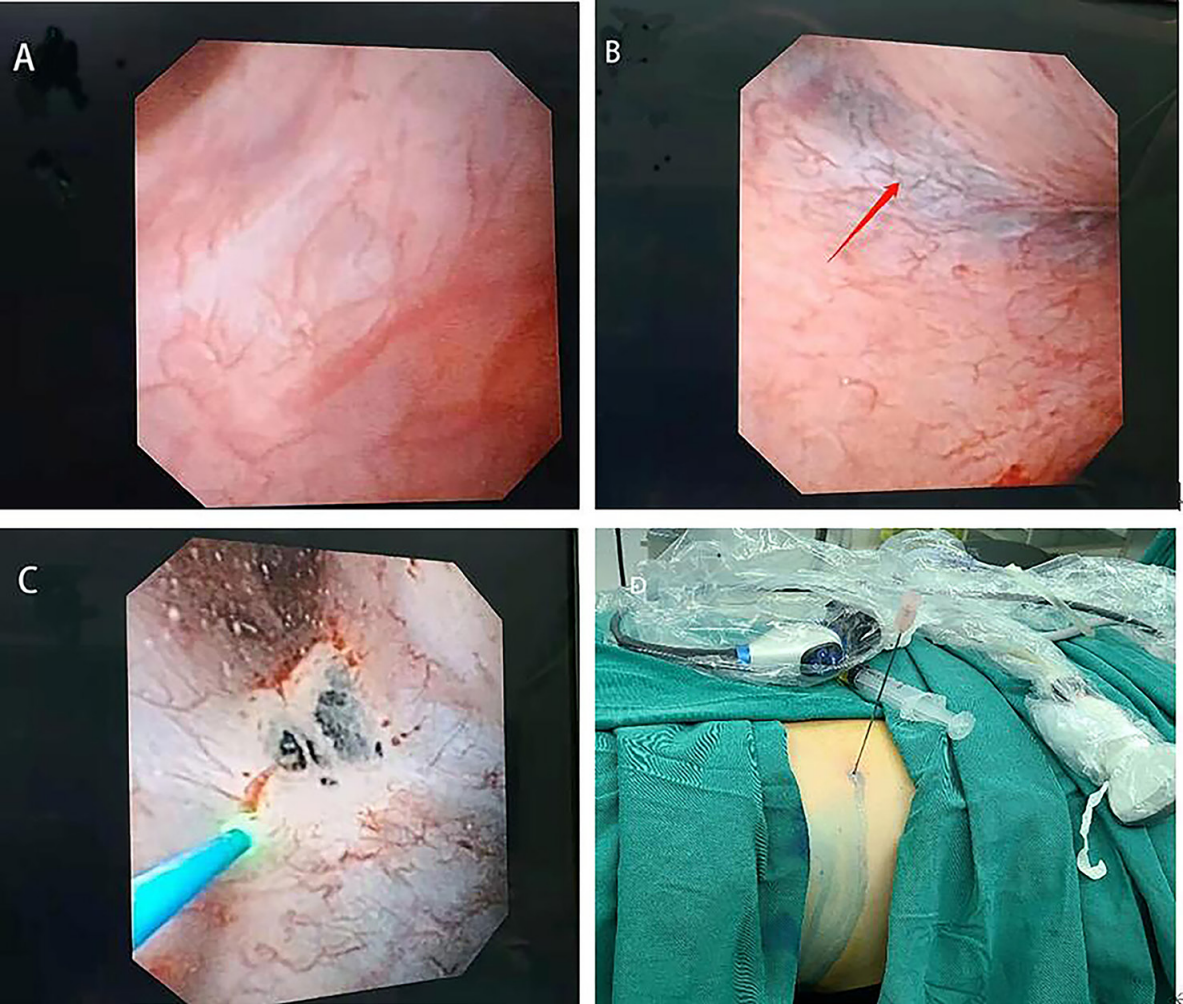
The left parapelvic cyst was identified by injecting methylene blue. The cyst wall before the injection of methylene blue is shown (A). Methylene blue was injected into the cyst to identify the parapelvic cyst, and the cyst wall (red arrow) became blue (B). The parapelvic cyst was incised by a holmium laser (C). Percutaneous renal puncture (D).

A total of 31 patients (Group 1) underwent a transurethral flexible ureteroscopic incision and drainage with holmium laser. A ureteral double J tube was routinely indwelled to dilate the ureter 2 weeks preoperatively. The procedure was performed under general anesthesia in the lithotomy position. To examine the ureter, a rigid ureteroscope (F8.0/9.8 Wolf) was retrogradely inserted into the renal pelvis, and a ureteral access sheath (Flexor 12/14F, Cook) was placed along the guide wire. The flexible ureteroscope (URF-V, OLYMPUS) was then inserted into the renal pelvis through the ureteral access sheath. A translucent blue area was observed on the mucosa of the renal pelvis and calyx, which was considered the area of renal cysts adjacent to the renal pelvis. The incision and drainage of the parapelvic cysts were performed using a holmium laser device (LUMENIS Versa Pulse Power Suite). The laser setting was 0.8 J with a frequency of 30 Hz (Figure-1). Percutaneous renal puncture was conducted by ultrasonography localization if the surgeon had difficulty identifying cysts. Methylene blue was injected into the cyst, which caused the cyst wall to turn blue, allowing the surgeon to properly identify the cyst wall (Figure-2). Finally, ureteral stenting (JJ stent) was routinely indwelled with proximal end inside the cyst for 4 weeks. There were five procedures (16%) in which the surgeon required methylene blue injection to locate cysts in this study.

In Group 2, 34 patients underwent retroperitoneal laparoscopic unroofing by the retroperitoneal approach. The procedure was performed in the standard left/right lateral decubitus position under general anesthesia. First, three functioning ports were installed (0.5, 0.5, and 1.0 cm, respectively). The kidneys were then isolated, especially the area adjacent to the cyst's location. The cyst was unroofed 0.5 cm adjacent to the renal parenchyma. The cystic wall was delivered to a pathologist for examination. A drainage tube (22 French) was inserted into the retroperitoneum.

### Statistical Analysis

Data are presented as the mean ± standard deviation (SD) or number. Age, BMI, cyst size, surgery duration, blood loss, length of hospitalization and cyst size at 1 year after the procedure were normally distributed. Student's t-test was used to compare continuous variables between groups, and the Chi-square test was used to compare categorical variables. SPSS 17.0 (SPSS, Chicago, IL, USA) was utilized for statistical analysis. Significance was established at P < 0.05.

## RESULTS

A total of 65 patients were successfully treated with transurethral flexible ureteroscopic incision and drainage or retroperitoneal laparoscopic unroofing. Among them, 31 with parapelvic cysts (Group 1) underwent a transurethral flexible ureteroscopic incision and drainage with holmium laser, while the remaining 34 patients (Group 2) underwent the retroperitoneal laparoscopic unroofing. When the surgeon could not locate the cyst wall during the transurethral flexible ureteroscopic incision and drainage technique in Group 2, retroperitoneal laparoscopic unroofing was performed instead. A comparison of clinical and perioperative factors between the transurethral flexible ureteroscopic incision and retroperitoneal laparoscopic unroofing is shown in Table-1.

The mean ages were 47.6±8.7 and 46.8±7.8 years in Groups 1 and 2, respectively, without a significant difference between the two groups (p = 0.701). No significant difference in gender was observed between Group 1 and Group 2 (p = 0.73). In terms of BMI, no significant difference was found between two groups (23.8±2.2 vs. 24.1±2.0 kg/m^2^, *p*=0.551). No significant difference regarding the location of renal cyst was observed between the two groups (*p*=0.271). Ultrasonography and CT were preoperatively performed to measure the size of renal cyst. The size of the renal cysts was 5.3±0.9 min in Group 1 and 5.1±0.9 min in Group 1, with no significant difference between the two groups (*p*=0.333). According to CT diagnosis, there were 31 patients with Bosniak category I renal cysts in Group 1, 33 with Bosniak category I renal cysts and 1 with a Bosniak category II renal cyst in Group 2, without a significant difference between Group 1 and Group 2 (*p*=0.336).

Group 1 had a shorter surgery duration than Group 2, and the difference between the two groups was statistically significant (30.1±4.3 vs. 54.4±6.4 min, p<0.001). Blood loss was 5.5±1.7 mL and 59±9.9 mL in Groups 1 and 2, respectively, with a significant difference between the two groups (p<0.001). The length of hospitalization in Group 1 was shorter than that in Group 2, with statistically significant difference between the two groups (4.5±0.8 vs. 5.6±0.9 days, p<0.001). The follow-up examination was postoperatively performed to measure the size of the renal cyst. At one year following the procedure, there was no significant difference in cyst size between the two groups (1.0±0.9 vs. 0.6±0.6 cm, *p*=0.106). No severe complications were observed in the two groups. In Group 1, significant hemorrhage was noted in 1 patient, which lasted for 2 days after the procedure. One patient had transient fever (38.7 °C temperature) in Group 2, but no significant difference was found between the two groups (*p*=0.947).

## DISCUSSION

The kidney is prone to cystic lesions. A parapelvic cyst is a cyst that occurs near the renal pelvis or pedicle, and its occurrence incidence increases with age. Parapelvic renal cysts manifest as a result of a special type of renal cystic disease (2), are nonhereditary, and cysts that develop adjacent to the renal pelvis are especially serious. Kiryluk (3) describes renal cysts that originate from the renal sinus, around the renal pelvis or renal sinus as parapelvic cysts. Chronic inflammation, according to Kutcher et al. (6), created parapelvic cysts by causing localized growth of pelvic lymphatic vessels.

Parapelvic cysts are frequently asymptomatic because they grow slowly. Parapelvic cysts cause symptoms by compressing the renal collecting system and renal vessels. The common symptoms include lumbar pain, hypertension, hematuria, recurrent urinary tract infection, and urinary tract obstruction (7, 8). Parapelvic cysts need surgical intervention when larger cysts cause symptoms.

To date, various methods have been used for the treatment of renal cysts, including percutaneous sclerotherapy, unroofing by open surgery, laparoscopic unroofing, and ureteroscopic drainage. Compared with simple renal cysts, the treatment of parapelvic cysts is relatively difficult due to the cyst's adjacent location to renal pelvis and vessels (9, 10). Percutaneous sclerotherapy is simple and economical. However, the recurrence rate for cysts is high due to the existence of a cyst wall. In addition, because the parapelvic cyst is adjacent to the renal hilum and pelvis, sclerotherapy could cause severe pyelonephritis or secondary ureteropelvic junction obstruction (11–13). In the past, laparoscopic unroofing was the preferred treatment for parapelvic cysts. Most surgeons, however, considered laparoscopic unroofing challenging. Because of the deep position of parapelvic cyst, the renal pelvis and vessels are easily injured intraoperatively (14, 15). The study reported that the incidence of pelvic injury was 9.5% during laparoscopic unroofing (8).

With the development of minimally invasive technology, Basiri et al. (15–18) reported that ureteroscopic incision and internal drainage were used to treat parapelvic cysts. In 1991, Kavoussi et al. (19) reported that they successfully performed ureteroscopic incision and internal drainage by ureteroscopy. They considered that this method has the advantages of minimal invasiveness, less postoperative pain, and rapid recovery. In this study, 31 patients successfully underwent a transurethral flexible ureteroscopic incision and drainage with holmium laser. Under the flexible ureteroscope, the visual field was not limited, the pelvis and all calyces were observed. The flexible ureteroscope can reach the target calyces and incise parapelvic cysts. Compared with the method of retroperitoneal laparoscopic unroofing, no significant difference was observed in terms of cyst size at one year postoperatively.

The key to ureteroscopic incision and drainage is to locate and identify renal cysts under a flexible ureteroscope. To avoid renal parenchyma or renal vessel injury, the incision should be located in the thin wall of the parapelvic cyst. The typical wall of a parapelvic cyst looks transparent. However, the surface of some parapelvic cysts is the same as that of the renal pelvis; therefore, it is difficult to identify the parapelvic cysts under the flexible ureteroscope (20). When methylene blue is injected into the cyst, the cyst wall turns blue, which can aid surgeons accurately identify the cyst wall (21). For parapelvic cysts in the posterior part of the kidney, percutaneous renal puncture was performed under B-ultrasound, the puncture needle was inserted into the renal pelvis through the cyst, and then an incision was performed along the puncture. Ulisses L G Pereira Sobrinho (22) reported that surgeon can train before proposing the appropriate surgical schedule to the patient using the 3D printed kidney. In this study, only one patient failed to undergo transurethral flexible ureteroscopic incision and drainage because the surgeon could not identify the cyst wall.

Some limitations exist in this study. First, selection bias occurred in our study due to its retrospective nature. Second, because this was a single center study, the number of patients was rather small, and further prospective randomized research is needed. Third, the transurethral flexible ureteroscopic incision and drainage has a number of drawbacks, such as higher costs and the need for two hospitalizations. Despite the limitations described above, the results of our study suggest potential avenues for future research and possible practice changes.

## CONCLUSIONS

In summary, transurethral flexible ureteroscopic incision and drainage with holmium laser in the treatment of parapelvic renal cysts has obvious advantages over traditional surgery, and is worthy of advancement and application, but its long-term effect needs further follow-up studies.

## ABBREVIATIONS

CT =: enhanced computed tomography
SD =: standard deviation
BMI =: indicates Body Mass Index
